# Clinical presentation, demographics and outcome of Tuberculosis (TB) in a low incidence area: a 4-year study in Geneva, Switzerland

**DOI:** 10.1186/1471-2334-9-217

**Published:** 2009-12-31

**Authors:** Omar Kherad, François R Herrmann, Jean-Pierre Zellweger, Thierry Rochat, Jean-Paul Janssens

**Affiliations:** 1 Department of Internal Medicine; Geneva University Hospitals and University of Geneva, Geneva, Switzerland; 2 Department of Rehabilitation and Geriatrics; Geneva University Hospitals and University of Geneva, Geneva, Switzerland; 3 Swiss Lung Association, Berne, Switzerland; 4 Division of Pulmonary Diseases; Geneva University Hospitals and University of Geneva, Geneva, Switzerland

## Abstract

**Background:**

The incidence of tuberculosis (TB) in developed countries has decreased since the 1990s, reflecting worldwide efforts to identify and treat TB according to WHO recommendations. However TB remains an important public health problem in industrialized countries with a high proportion of cases occurring among subjects originating from high prevalence countries. The aim of this study was to describe clinical and social characteristics of patients with TB and their outcome in a low incidence area with a high immigration rate.

**Methods:**

Four-year retrospective study based on a computerized database and subsequent review of medical records of all patients with TB followed at the outpatient section of the Division of Pulmonary Diseases, Geneva University Hospital, Switzerland.

**Results:**

252 patients (84% foreigners, 25% asylum seekers) aged 38 ± 19 yrs were studied (11% co-infected with HIV). TB was intrapulmonary (TBP) in 158 cases (63%), extrapulmonary (TBE) in 137 (54%), and both in 43 cases (17%). TBP was smear (S)+/culture (C)+ in 59%, S-/C+ in 37%, S-/C- in 4%. Smoking was significantly associated with cavitary disease.

Time from onset of symptoms to diagnosis was 2.1 ± 3.1 months. Initially, 10% were asymptomatic; 35% had no general symptoms. Despite systematic sputum analysis (induced or spontaneous), TBP was confirmed only by bronchoscopy in 38 subjects (24% of TBP). Side effects requiring changes in treatment occurred in 38 cases (11%).

Treatment was completed in 210 (83%) patients. In 42 cases, follow up was unsuccessful; causes were: failure (n = 2; 0.8%), defaulters (n = 8; 3%), transfer out (n = 28; 11%) and death (n = 4; 1.6%). Relapse rate was 0.24 per 100 patient-years. Considering S+ TBP only, success rate was 87%.

**Conclusion:**

TB in our area is predominantly a disease of young foreign-born subjects. Smoking appears as a possible risk factor for cavitary TBP. Time to diagnosis remains long. Compliance to treatment is satisfactory. Success rate for S+ TBP is within WHO objectives.

## Background

With an estimated 9 million new cases and 2 million deaths every year, tuberculosis (TB) remains a leading public health problem worldwide [1]. In industrialized countries, incidence of TB has been regularly decreasing since the 1990s, although recently, several European countries have reported a slight increase in TB, mostly related to young immigrants from high-incidence countries [2,3].

In Switzerland, incidence of TB is low (8.5 per 100 000 population). Over the past 10 years, as in most countries of Western Europe, the proportion of indigenous TB cases has continuously decreased while that of foreigners (presently 76%) has increased. In recent years, the overall incidence of tuberculosis in Switzerland has stabilized due to immigration from high prevalence countries [4].

In 1998, the World Health Organization (WHO) and the International Union Against Tuberculosis and Lung Disease (IUATLD) published recommendations standardising the evaluation of treatment outcome for TB in Europe [5]. Outcome targets set by WHO were to achieve at least an 85% cure rate and 70% detection of smear positive TB. Based on a recent meta-analysis, only 75% of TB cases are successfully treated in Europe, but with a very high heterogeneity in results [6]. The only 3 studies performed in Switzerland cover 1991 and 1998, and reported a successful outcome in a rather low 79% of TB cases [7-9].

The Geneva area offers a privileged opportunity to study treatment outcome in a population with a low TB incidence, as almost all cases of TB are supervised by one specialized centre.

The aim of this study is thus to describe recent clinical and social characteristics of patients with TB, to analyse treatment outcome and to identify factors associated with unsuccessful outcome.

## Methods

In the Canton of Geneva (450,000 residents), incidence of TB is 2.5 times above the national average (20 cases per 100,000 inhabitants), mainly because of a higher proportion of foreign-born residents (45% in 2008). Most TB cases are either diagnosed at Geneva University Hospital (emergency ward, outpatient or inpatient clinics) and referred to the outpatient clinic of the Division of Pulmonary Diseases, or directly referred to the Division of Pulmonary Diseases by private practitioners for treatment and follow-up. Albeit for asylum seekers, there is no systematic screening program for TB in our area. All patients treated for TB are entered in a computerized database which stores information on gender, age, origin, microbiological details, co-morbidities including results of HIV testing (performed in all cases after informed consent), diagnosis of previous TB, antimicrobial drug resistance, chest X-ray findings and treatment prescribed.

Medical records of all cases of TB for whom treatment was started at our centre between 1.1.1999 and 31.12.2002 were reviewed; compliance and tolerance to treatment (monthly ASAT: Aspartate amino-transferase; and ALAT: Alanine amino-transferase, reported side-effects), treatment interruptions and their causes were analyzed. We excluded cases which were not confirmed by positive culture or histopathology (n = 30).

Treatment regimens for TB were in agreement with international guidelines. Default treatment for pulmonary TB was a four-drug regimen of isoniazide, rifampicine, ethambutol and pyrazinamide for 2 months followed by 4 months of isoniazide and rifampicine only. Treatments were administered on a daily basis, or, in case of DOT, 3 times weekly after at least 2 weeks of daily treatment. Duration of treatment was extended to 12 months in case of osteo-articular TB, or central nervous system involvement. Incidence of primary resistance to isoniazide in our area is 4.9% [4]. For patients with smear positive sputum samples, treatment was initiated during a 2 week hospital stay, and then pursued on an outpatient basis. The decision to implement DOT (Directly Observed Therapy) was left to the discretion of attending physicians. Compliance was assessed by attendance to monthly visits and urinary testing for isoniazid (INH). HIV testing was performed in all patients with TB.

For patients with suspected pulmonary TB, a trial of induced sputum production was systematically performed by chest therapists whenever spontaneous sputum samples could not be obtained; when microscopic examination of samples for acid-fast bacilli (AFB) was negative, or adequate samples could not be obtained, bronchoscopy was performed. During the procedure, bronchoalveolar lavage (BAL), and bronchial aspirate (BA) were performed and analysed systematically. Brushings or biopsies were performed only if indicated by endoscopic findings. Post-bronchoscopy sputum samples were not systematically analysed.

The Ethics Committee of Geneva University Hospital approved the study protocol.

### Definitions

*Outcome of treatment *is reported as follows in agreement with WHO and IUATLD criteria [5]:

1/successful outcome: treatment completed and assumed cure with or without microbiological evidence of cure; 2/unsatisfactory outcome: treatment failure, defaulters, death or transfer out. The latter category includes patients who left the country (expelled or not), patients transferred within Switzerland to another hospital, and patients followed by a private physician through which information on treatment outcome could not be obtained.

Following WHO recommendations [5], analysis of outcome was performed separately for new cases and retreatment cases.

*Serious drug-related side effects *were defined as those requiring a change in therapy or hospitalization. Biological hepatitis was defined as an elevation of ASAT and/or ALAT at least 5 times above upper limit of normal values.

We defined as *general symptoms*: fever, weight loss or nocturnal sweating. *"Any drug resistance" *refers to resistance to one of the first line therapy drugs (isoniazide: INH, rifampicine, ethambutol, pyrazinamide) while *multi-drug resistance (MDR) *is defined by simultaneous resistance to at least INH and rifampicine.

### Statistical analysis

Variables are reported as mean ± standard deviation (SD). Comparison between groups was performed using unpaired t tests, chi-square tests or Fischer's exact test when appropriate. Multiple logistic regressions were performed to analyse the relationship between independent variables selected by a stepwise backward analysis and specific dependant variables. The results are presented as odds ratios (OR) with 95% confidence intervals (95%CI). P values < 0.05 were considered statistically significant. Statistical analyses were performed with Stata Statistical Software for PC computers (version 10.1, 2007; Stata Corporation, College Station, Texas, USA).

## Results

### Patients

Between January 1999 and December 2003, 252 patients (120 male: 47%; 132 female: 53%; aged 38 ± 19 yrs; range: 15-92) were eligible and included in the present study. This represents 78.5% of all cases of TB treated in the Geneva area during the same period (irrespective of bacteriological confirmation). Of these 252 patients, 84% were foreign-born. Socio-demographic characteristics of patients and risk factors for TB are listed in table 1.

**Table 1:** Socio-demographic characteristics of patients

n = 252	n (%)
**Ethnic origin**	
• Caucasian	98 (39)
• Asian	20 (8)
• Hispanic	39 (15)
• Black	95 (38)
**Socioeconomic status**	
• Asylum seekers	66 (26)
• Political refugees	18 (7)
• Illegal status	12 (5)
• Homeless	2 (1)
**Immunosuppression**	
• Co-infection with HIV	28 (11)
CD4 lymphocyte count (/mm^3^)	
◦ > 500	5 (18)*
◦ 200 - 500	5 (18)*
◦ 50 - 200	10 (36)*
◦ < 50	8 (28)*
• **Immunosuppressive treatment**	**3 (2)**
**Substance abuse**	
• Active smokers	46 (18)
• Alcohol abuse	16 (6)
• Drug abuse	6 (2)
Prior TB	10 (4)

HIV = Human immune-deficiency virus; TB = tuberculosis.

*: percentage of HIV-infected subjects.

**Figure 1:**
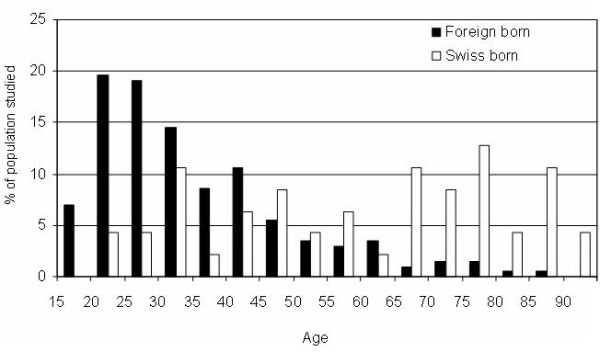
**Distribution of TB cases according to age and origin**. Dark bars: foreign-born (n = 212, 84%); white bars: indigenous (Swiss-born) population (n = 40, 16%).

Foreign-born patients were on average younger (35.1 ± 15.0 yrs, range: 15-88) than indigenous patients (62.5 ± 19.3 yrs, range 23-92) (Figure 1). However, figure 1 shows a bi-modal distribution of indigenous patients with a group of younger patients (n = 17, aged < 50 years) which had a very high rate of HIV co-infection (47%) when compared to foreign-born subjects of the same age group (n = 164, 11.6%, p = .001; Fisher's exact test)

Foreign-born patients originated mainly from Africa (38.4%), Europe (19.3%), Latin America (8.5%), and Asia (9.5%). Fifteen cases (6%) were diagnosed through systematic screening of asylum seekers at the border (chest X-ray). Only 4 cases (1.5%) were detected in contact-tracing procedures (0.2% of 2095 subjects screened)[10]. Twenty-eight cases (11%) were co-infected with HIV: only 3 were on antiretroviral therapy at diagnosis (Table 1).

### Clinical presentation

Clinical presentation is summarized in Table 2. TB was pulmonary (TBP) in 158 cases (63%) (TBP only: n = 115, 46%), extra-pulmonary (TBE) in 137 cases (54%) (TBE only: n = 94, 37%), and both (TBP+TBE) in 43 cases (17%). More than half of the extra-pulmonary cases had adenitis (n = 74, 54%).

**Table 2:** Clinical presentation

n = 252	n (%)
Pulmonary TB	158 (63)
• Smear +/Culture +	93 (59)*
• Smear -/Culture +	58 (37)*
• Smear -/Culture -	7 (4)* ^#^
• Cavitary disease	36 (23)*
• New TB cases	151 (96)*
Extrapulmonary TB	137 (54)
• Adenitis	74 (29)
• Pleuritis	18 (7)
• Abdominal TB	17 (7)
• Osteo-articular TB	17 (7)
• Urogenital tractus TB	16 (6)
• ENT TB	4 (2)
• Other	21 (8)

TB: tuberculosis; ENT = ear, nose and throat;

*: percentage of pulmonary TB cases; sputum or bronchoscopy samples.

Total number of cases exceeds 252 because multiple organ involvement possible.

^#: ^Smear -/Culture -: diagnosis based on either positive culture or histopathology of other organ, and favourable radiological evolution of pulmonary involvement under treatment

Table 3 describes the clinical presentation of TB according to socio-demographic characteristics of patients. The odds ratio of cavitary TB was markedly increased in smokers (almost six times that of non-smokers), and decreased in older subjects. Occurrence of extra-thoracic TB was associated with female gender but did not differ between foreign-born and indigenous patients. HIV co-infection, and, to a lesser degree, alcohol dependence and female gender were significantly associated with disseminated TB.

**Table 3:** Adjusted odd ratios for clinical presentation of TB according to socio-demographic characteristics of patients

	Extra-pulmonary Tb		Cavitary Tb		Disseminated Tb	
	OR	95% CI	OR	95% CI	OR	95% CI
Age						
▪ < 35 y	1.00	-	1.00	-	1.00	-
▪ 35-60 y	1.80	[0.91-3.55]	0.24	[0.08-0.73]	0.80	[0.31-2.07]
▪ ≥ 60 y	1.48	[0.57-3.90]	**0.13**	**[0.03-0.62]**	0.69	[0.15-3.30]
Male gender	**0.38**	**[0.20-0.70]**	0.68	[0.28-1.64]	**0.31**	**[0.12-0.81]**
Ethnic origin						
▪ Caucasian	1.00	-	1.00	-	1.00	-
▪ Asian	2.18	[0.62-7.65]	0.72	[0.12-4.26]	1.79	[0.28-11.36]
▪ Hispanic	1.48	[0.51-4.25]	0.82	[0.21-3.18]	1.34	[0.26-6.95]
▪ Black	1.47	[0.59-3.71]	0.76	[0.23-2.48]	1.34	[0.30-5.94]
Swiss born	1.05	[0.37-2.97]	2.93	[0.80-10.72]	0.91	[0.17-4.78]
Co-infected with HIV	2.20	[0.86-5.60]	0.11	[0.01-1.06]	**7.28**	**[2.45-21.60]**
Active smoker	**0.38**	**[0.14-1.02]**	**5.80**	**[1.85-18.17]**	0.88	[0.20-3.91]
Alcohol abuse	0.52	[0.10-2.65]	0.56	[0.10-3.04]	*	*

CI = confidence interval; OR = odd ratio,* dropped: predicts failure perfectly

### Diagnosis and diagnostic procedures

Mean time to diagnosis (i.e. time elapsed between first symptoms recalled by the patient and beginning of treatment) was 2.1 ± 3.1 months (range: 0-12, Figure 2) without any statistical difference between the different groups in terms of socio-demographic and clinical characteristics in multivariate analysis. At diagnosis, of all patients, 10% were asymptomatic and 35% had no general symptoms.

**Figure 2:**
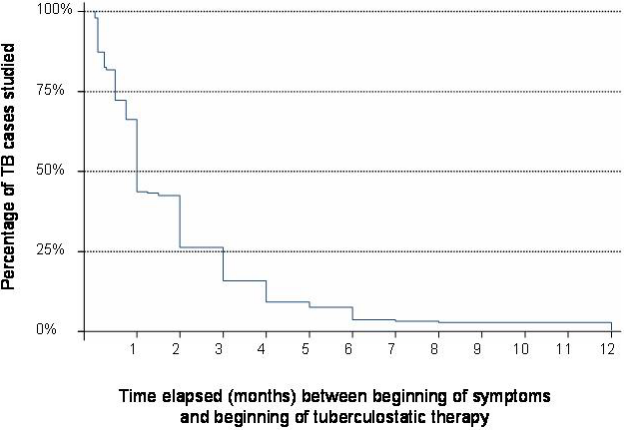
**Kaplan Meier curve describing time to diagnosis: time elapsed (in months) between beginning of symptoms and beginning of antituberculosis therapy**. One case is not represented here: it was a young female patient who was unsuccessfully treated with several antibiotics for slowly evolving atypical cutaneous lesions until a biopsy was performed after 24 months yielding a diagnosis of TB.

**Figure 3:**
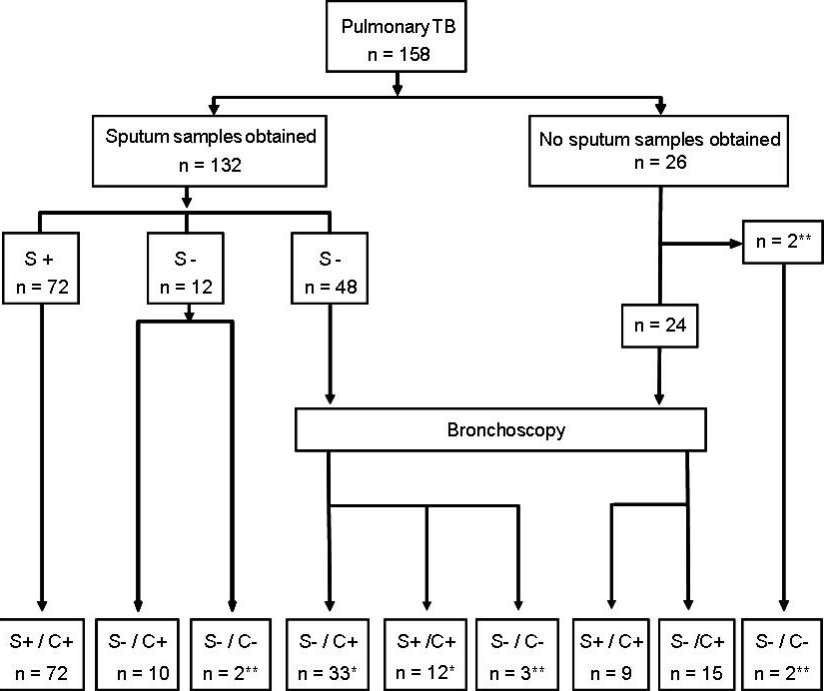
**Diagnostic procedures for bacteriological confirmation of tuberculosis in 158 cases of pulmonary TB**. 113 cases were diagnosed through sputum analysis: 72 S+/C+ and 41 S-/C+. *: of 48 subjects who had bronchoscopy because of S- sputum samples, 31 had positive sputum cultures; for 14 subjects, *only *samples obtained by bronchoscopy were culture positive; 12 bronchoscopy samples were S+. **: S-/C- cases; diagnosis was confirmed in these cases either by histo-pathological examination or culture of non-respiratory samples.

**Figure 4:**
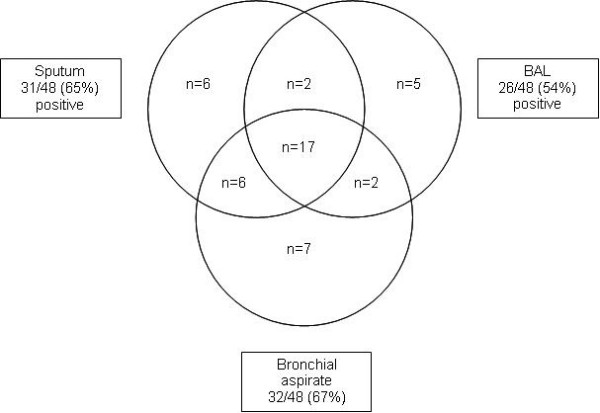
**Results of cultures for mycobacteria of sputum samples, broncho-alveolar lavage (BAL) and bronchial aspirate (BA) in n = 48 subjects in whom bronchoscopy was performed because of negative microscopic analysis of sputum smears**. Three cases with negative culture of sputum, BAL and BA are not shown.

Figures 3 and 4 depict the relative contribution of sputum and bronchoscopy for confirming the diagnosis of pulmonary TB. All positive cultures were confirmed by PCR. Bronchoscopy was performed in 24 of 26 subjects in whom adequate sputum samples could not be obtained and in 48 of the 59 sputum S- patients.

Among the 158 cases of pulmonary TB, most cases (n = 113, 71.5%) were diagnosed through sputum analysis (smear+ (S+)/culture+ (C+): 72, S-/C+: 41 (figure 3); 7 cases were S-/C- (diagnosis was confirmed by extra-pulmonary sampling). In 38 cases (24% of pulmonary TB: 24 patients in whom no sputum samples could be obtained and 14 of the 48 S- patients), bacteriological confirmation of pulmonary TB was obtained *only *by bronchoscopy (bronchial aspirate: BA, and/or broncho-alveolar lavage: BAL). Furthermore bronchoscopy samples were S+ in 12 sputum S- cases (diagnosis was confirmed by culture with both procedures): in these 12 cases, bronchoscopy was a time-saving procedure for confirming the clinical suspicion of TB.

For the 48 patients who had bronchoscopy after S- sputum, results of cultures for sputum, BA, and BAL are shown in Figure 4. Positive culture rate was slightly higher for BA (67%) and sputum (65%) than for BAL (54%).

### Treatment and side effects

Drug-related side effects were noted in 75 cases (30%); however, side effects severe enough to change or stop treatment were reported in only 38 cases (15%): in 2/3 of cases they were due to symptomatic liver test abnormalities which occurred mainly within the first two months of treatment (mean: 6.2 ± 7.3 weeks). Biological hepatitis (6%) was associated with female gender (OR 9.1; 95% CI: 1.50-50), co-infection with HIV (OR 11.1, 95% CI: 2.34-52.8) and alcohol abuse (OR 21.3; 95% CI: 1.6-283.8). Other side effects reported were mainly general symptoms (weight loss, vertigo, asthenia) (n = 20), rash (n = 17) and neurological symptoms (polyneuropathy, n = 10). Probability of having a serious side effect was five-fold higher in female patients (OR 4.76; 95% CI 1.85-12.5).

Resistance to at least one of the first line therapy drugs was reported in 20 cases (8%). A previous treatment for TB was highly associated with the risk of resistance (OR 13.6; 95% CI 2.02-91.9). Rate of multi-drug resistant TB (MDR-TB) was 2%: all five cases were foreign-born. By univariate analysis, MDR-TB was also associated with HIV co-infection and prior treatment for TB (p < 0.05). No XDR-TB (extensively drug-resistant) case was detected.

Treatment was administered as a direct observed therapy (DOT) in 47 cases (19%): multivariate analysis did not disclose any significant differences between DOT and non-DOT patients in terms of socio-demographic and clinical characteristics or outcome.

### Outcome

Table 4 summarizes causes of unsatisfactory outcome. Of the 252 subjects included, treatment was completed in 210 (83%) cases. In 42 cases, (17%) follow-up was unsuccessful. In multivariate analysis adjusted for age, sex, ethnic origin, HIV co-infection, socioeconomic status, substance abuse and clinical presentation (TBP, TBE), the only factor that was significantly associated with unsuccessful treatment outcome was male gender (OR 3.0; 95% CI 1.04-8.64). Conversely, asylum seekers had a higher probability of successful outcome (OR 0.05; 95% CI 0.01-0.46). All five cases of MDR-TB were considered cured.

**Table 4:** Outcome of TB treatment adapted from WHO and IUATLD criteria [5]

	Pulmonary C+ TB* n = 144 (%)	Pulmonary S+ TB* n = 68 (%)	†**All cases TB** n = 252 (%)
**Successful**	115 **(80)**	59 **(87)**	210 **(83)**
**Unsuccessful**	29 (20)	9 (13)	42 (17)
**Death**	4 (2.8)	1 (1.5)	4 (1.6)
**Treatment failure**	1 (0.7)	1 (1.5)	2 (1)
**Defaulters**	6 (4)	0 (0)	8 (3)
**Transfer out**	18 (13)	7 (10)	28 (11)
**Departure from country**	13	4	23
**Extradition**	3	2	3
**Follow-up by specialist or other hospital in Switzerland with no information available**	2	1	2

C+ = culture positive; S+ = smear positive

*new cases of pulmonary TB

†WHO criteria do not apply to extrapulmonary TB: however, results for all TB cases are shown for comparison

Relapse occurred in two cases; relapse rate was 0.24 per 100 patient-years occurring on average 2.5 ± 2.1 months after completion of treatment. As shown, fatality rate was 1.6% (n = 4); none of these patients had a post-mortem. Three cases were elderly patients with other co-morbidities which may have contributed to death.

According to WHO criteria, 87% of new cases of pulmonary TB with a positive sputum smear (n = 68) and 80% of those with a positive sputum culture (n = 144) had a successful outcome (Table 4); in these cases, a multivariate analysis adjusted for age, sex, ethnic origin, HIV co-infection, socioeconomic status, substance abuse and clinical presentation (TBP, TBE) did not identify any risk factor associated with an unfavourable outcome.

## Discussion

This study describes the clinical and social characteristics of patients with TB and their outcome over a 4-year period in a low incidence area for TB, with a high immigrant population. Our main findings are: 1/TB in our area is essentially a disease of young foreign-born subjects (84%). This proportion is well above the 40-60% of foreign-born subjects reported in most low-incidence countries. Indigenous cases are significantly older [11-14]; 2/Prevalence of HIV co-infection was higher than previously reported in our country (11%), and in Western Europe; 3/Extra-pulmonary TB (TBE) was frequent (54%), and, more specifically, lymphadenitis. Surprisingly, lymphadenitis was not associated with ethnic origin, age or HIV infection but occurred more frequently in females; 4/Interestingly, cavitary TB was strongly related to smoking history, and occurred less frequently in elderly patients; 5/Although most cases of pulmonary TB were diagnosed through sputum analysis, bacteriological confirmation of TB was obtained by bronchoscopy *only *in 38 subjects (24% of all pulmonary TB); 6/Side effects severe enough to change treatment (15% of all cases) occurred more frequently in female subjects; 7/Time to diagnosis remains disturbingly long. Finally, applying WHO criteria to new sputum smear positive pulmonary cases only, treatment success rate for the 1999-2003 period was 87%, i.e. above the 85% success target set by WHO.

The very high proportion of young foreign-born subjects among TB cases reported in our area reflects trends recently reported in several countries of Western Europe, and in Canada [15]. In Switzerland, the number of cases of tuberculosis among foreign-born subjects (64% in 2004) exceeds that of native subjects since 1994, and, although TB incidence in the indigenous population continues to decrease, the linear decline of tuberculosis notification observed since 1990 has markedly subsided since 2000 [4]. Similarly, between 2001 and 2005 in Norway, Denmark, Sweden, Ireland, and Great Britain, increase in TB among foreign-born subjects has been such that national TB notification rates have in fact increased [2,3].

In this study, prevalence of HIV co-infection (11%) was much higher than the 3% rate of HIV seropositivity reported in TB patients in the WHO European region [16]. This rate is most probably influenced by the important number of immigrants from areas of the world where rates of TB-HIV co-infection are very high [17,18]. Despite a higher risk of disseminated disease [19], outcome was not worse in subjects co-infected with HIV. An unexpected observation, when compared to previous national reports [4] is the presence of a substantial group of young Swiss-born TB cases (< 50 yrs of age) in whom HIV infection was highly prevalent (47%). National data estimate that only 2.6% of Swiss subjects with TB are co-infected with HIV, vs. 20% in the present study; this observation is consistent with an increase in TB/HIV co-infection recently reported in England and Wales [20].

In our study, smokers had a 6-fold increase in risk of developing cavitary TB; smoking was not associated however with a higher rate of smear positive sputum samples. Smoking affects the clinical manifestations of TB by notably increasing occurrence of upper zone involvement, cavitary and miliary disease, and rate of positive sputum cultures [21-23]. Suggested mechanisms include decreased immune response, and decreased production of TNFα by macrophages [24]. Down regulation of macrophage TNF-α in the lungs may render the patient more susceptible to the development of progressive and severe disease. This mechanism was already described with the use of anti-TNFα treatment [25].

Conversely, cavitary disease was less frequent in elderly subjects. Clinical and radiological presentation of pulmonary TB in older subjects is frequently atypical, with a more frequent involvement of lower and middle lobes, of miliary patterns, and, in several studies, less frequent cavitary lesions [26,27], although this latter observation remains controversial [28].

Bacteriological confirmation of TB was obtained by bronchoscopy *only *in 24% of all pulmonary TB. Several studies have provided evidence supporting the use of sputum induction in smear-negative pulmonary TB disease. This procedure is safe, has a high diagnostic yield and is more cost effective than bronchoscopy [29-31]. However, in industrialized countries, the role of bronchoscopy among patients with smear negative TB needs to be clarified. A recent paper studying prospectively in asylum seekers in Switzerland noted that the diagnostic yield is increased by bronchoscopy and concluded that this procedure, when available, is recommended for sputum smear negative TB [32]. Although the design of the study does not allow a formal evaluation of the yield of bronchoscopy, our results clearly suggest that bronchoscopy when available, is contributive for rapid bacteriological confirmation of diagnosis in sputum smear negative pulmonary TB.

A main endpoint of our study was the outcome of TB treatment (Table 4). For new cases of sputum smear positive pulmonary TB only, treatment success rate was 87%, *i.e*. just above the WHO set target of 85%, and compared favourably with results recently reported in the European Community, which ranged from 70 to 80% [6,11-15]. A recent study by Ditah et al. which focused on outcome of TB treatment in the United Kingdom, suggested that WHO outcome criteria were not adapted to low incidence industrialized countries [33]. The same authors proposed new criteria to analyse outcome among all patients with TB (pulmonary and extrapulmonary) from a clinical perspective. Using these criteria, the rate of satisfactory outcome for all TB cases would be closer to 85% instead of 83% (Table 4), thus within WHO targets; these results are the best reported in our country since 1998 [7-9].

A few limitations of this study must be mentioned:

The retrospective nature of the study is a methodological limitation although the fact that all patients followed at our institution (78.5% of all cases notified in Geneva during this period), were prospectively included in a computerized database, and were followed at one centre minimizes the risk of selection bias. Through systematic reviewing of patient files, the good quality of the database could be assessed. Outcome criteria follow international standards, thus permitting comparison with other countries [6,7,12-14,33]. For some associations and OR computations, confidence intervals were wide due to small sample size.

Finally, our protocol does not allow a retrospective assessment of whether sputum samples analysed were spontaneous or induced, and thus a separate analysis of the relative contribution of these samples. The vast majority of patients suspected of TB in our area are referred to the emergency ward of our hospital, or evaluated in a hospital ward. When spontaneous sputum was not obtained, sputum induction was systematically performed by chest therapists, but samples processed by the laboratory do not bear the mention of how they were obtained.

## Conclusion

This study illustrates the present situation of TB in a low incidence industrialized country: TB patients are mostly young, foreign-born, with a high incidence of extra-thoracic involvement (mainly adenitis). Rate of co-infection with HIV was higher in Switzerland than reported in the WHO European region, and is a major risk factor for the younger indigenous cases described. For smear positive pulmonary TB, the 85% WHO target for successful outcome has been reached.

With one third of patients who do not report suggestive general symptoms, diagnosis of TB remains a challenge for clinicians: indeed time to diagnosis remains disturbingly long. The strong association between cavitary TB and smoking, and the contribution of bronchoscopy for smear negative TB warrant further investigation.

## Competing interests

The authors declare that they have no competing interests.

## Authors' contributions

OK, JPJ, and TR were involved in the overall study design and protocol development. OK, JPJ and FH were responsible for data management and oversaw data analysis. OK, JPJ and JPZ participated in writing the manuscript, which all authors have reviewed and approved.

## Pre-publication history

The pre-publication history for this paper can be accessed here:

http://www.biomedcentral.com/1471-2334/9/217/prepub
